# Polymorphism in COMT is associated with IgG_3_ subclass level and susceptibility to infection in patients with chronic fatigue syndrome

**DOI:** 10.1186/s12967-015-0628-4

**Published:** 2015-08-14

**Authors:** Madlen Löbel, Agnes Anna Mooslechner, Sandra Bauer, Sabrina Günther, Anne Letsch, Leif G Hanitsch, Patricia Grabowski, Christian Meisel, Hans-Dieter Volk, Carmen Scheibenbogen

**Affiliations:** Institute for Medical Immunology, Charité-Universitätsmedizin Berlin, Campus Virchow, Augustenburger Platz 1/Südstraße 2, 13353 Berlin, Germany; Immunology Department, Labor Berlin GmbH, Charité University Medicine Berlin, Campus Virchow, Berlin, Germany; Department of Hematology, Oncology, Charité Campus Benjamin Franklin, Berlin, Germany; Berlin-Brandenburg Center for Regenerative Therapies (BCRT), Charité University Medicine Berlin, Berlin, Germany

**Keywords:** Immunoglobulins, HPA axis, Chronic fatigue syndrome

## Abstract

**Background:**

Chronic fatigue syndrome (CFS) is considered as a neuroimmunological disease but the etiology and pathophysiology is poorly understood. Patients suffer from sustained exhaustion, cognitive impairment and an increased sensitivity to pain and sensory stimuli. A subset of patients has frequent respiratory tract infections (RRTI). Dysregulation of the sympathetic nervous system and an association with genetic variations in the catechol-O-methyltransferase (COMT) and glucocorticoid receptor genes influencing sympathetic and glucocorticoid metabolism were reported in CFS. Here, we analyzed the prevalence of SNPs of COMT and glucocorticoid receptor-associated genes in CFS patients and correlated them to immunoglobulin levels and susceptibility to RRTI.

**Methods:**

We analyzed blood cells of 74 CFS patients and 76 healthy controls for polymorphisms in COMT, FKBP5 and CRHR1 by allelic discrimination PCR. Serum immunoglobulins were determined by immunoturbidimetric technique, cortisol levels by ECLIA.

**Results:**

Contrary to previous reports, we found no difference between CFS patients and healthy controls in the prevalence of SNPs for COMT, FKBP5 and CRHR1. In patients with the Met/Met variant of COMT rs4680 we observed enhanced cortisol levels providing evidence for its functional relevance. Both enhanced IgE and diminished IgG_3_ levels and an increased susceptibility to RRTI were observed in CFS patients with the Met/Met variant. Such an association was not observed in 68 non-CFS patients with RRTI.

**Conclusion:**

Our results indicate a relationship of COMT polymorphism rs4680 with immune dysregulation in CFS providing a potential link for the association between stress and infection susceptibility in CFS.

## Background

Chronic fatigue syndrome (CFS) is a severe disease characterized by persistent exhaustion, cognitive dysfunctions and flu-like symptoms leading to a substantial reduction of quality of life [1]. In a subset of CFS patients there is an acute onset of the disease with an infection. Further, a subset of patients suffers from recurrent respiratory tract infections (RRTI) [2]. A hallmark of CFS is aggravation of symptoms by stress [3, 4]. Most patients suffer from severe cognitive impairment [5] and increased sensitivity to pain and sensory stimuli [6]. Dysregulation of autonomic nervous system, an enhanced sensory processing in the central nervous system (CNS) and dysfunction of the hypothalamic–pituitary–adrenal (HPA) axis are considered as pathophysiological mechanisms in CFS [7]. Enhanced epinephrine and norepinephrine levels were found in CFS patients in some [8, 9] but not all studies [10, 11]. Although adrenocorticotropic hormone primarily controls the release of cortisol, also enhanced levels of the catecholamine norepinephrine lead to the release of cortisol [12]. Diminished and enhanced cortisol levels were reported in CFS patients [13–16]. Further, there are studies showing that the regulation of the immune function by cortisol and catecholamines is dysregulated in CFS patients [10, 17]. In particular, an increased sensitivity of glucocorticoid receptors on lymphocytes is proposed to induce a Th2 shift in CFS patients [18, 19]. A resistance of T cell function to glucocorticoid and β2-adrenergic agonists is reported by others [10].

Presently, few small studies show that CFS is associated with genetic variations in glucocorticoid receptor associated genes and the catechol-O-methyltransferase (COMT), an enzyme responsible for the inactivation of the catecholamines norepinephrine, epinephrine and dopamine by methylation [20]. For the A allele of the COMT single nucleotide polymorphism (SNP) rs4680 at codon 158, resulting in a substitution of Val with Met, reduced enzyme activity was shown [21]. Higher frequencies of this variant was shown in CFS patients [22, 23] and in fibromyalgia [24]. In associative studies an increased occurrence of stress-related disorders and neuronal stress response including enhanced cortisol levels are published for polymorphisms of the corticotropin-releasing hormone receptor 1 (CRHR1) and FK506 binding protein 5 (FKBP5) that interacts with corticoid receptor complexes [25–28].

Within our study, we investigated the prevalence of the SNPs for COMT rs4680, FKBP5 rs1360780 and CRHR1 rs12944712 in CFS patients

## Patients and methods

### Human blood samples

Patients were recruited from the outpatient clinic for immunodeficiencies at the Institute for Medical Immunology at the Charité Universitätsmedizin Berlin between 2011 and 2014. CFS patients submitted were not preselected for an infection history or immunodeficiency. Patients were of Caucasian ethnicity and diagnosed with CFS by fulfilling both Canadian and Fukuda criteria [29] and exclusion of other medical or neurological diseases or depression. Patients with chronic systemic steroid or immunosuppressant therapy or a diagnosis of primary immunodeficiency were excluded from this study. Age and sex-matched healthy controls were recruited from staff. Enhanced susceptibility to infection was defined as having a history of at least four respiratory tract infections per year or a history of severe infections (e.g. pneumonia). Reports on history of RRTI were assessed by clinical staff and in two patient questionnaires. Data was analyzed by an independent reviewer. A further group of patients was included who suffered from RRTI but had neither CFS nor immunodeficiency. IgG_3_/_4_ subclass deficiency was not an exclusion criterion for recruitment in this study. Immunoglobulin and cortisol levels were analyzed in the routine diagnostics laboratory of the Charité, Labor Berlin GmbH according to their standards, which require immediate transport to the laboratory which is usually below 1 h and immediate processing. One sample was used to prepare DNA in our research laboratory for the SNP analyses and DNA quality was checked at 260/280 ratio at the nanodrop spectrophotometer. The study was approved by the Ethics Committee of Charité Universitätsmedizin Berlin in accordance with the 1964 Declaration of Helsinki and its later amendments. All patients and healthy controls gave informed consent.

### Quantification of immunoglobulins and cortisol

Serum immunoglobulins G and E were determined at Labor Berlin GmbH by immunological turbidity test (Roche Diagnostics). IgG subclasses were measured by immunoturbidimetric technique (The Binding Site) and cortisol levels by ECLIA (Cobas 8000, Roche). The patient’s serum cortisol was measured between 9 am and noon depending on the time of visit at our out-patient clinic, but we assume equal distribution of blood drawing in all haplotype groups.

### SNP analysis

Analysis of the SNPs COMT rs4680 and FKBP5 rs1360780 was performed according to manufacturer’s instruction for the Applied Biosystems 7300/7500/7500 Fast Real-Time PCR System on 10 ng of genomic DNA. Probes were purchased from Applied Biosystems.

### Statistical analysis

Statistical data analysis was done using the software SPSS Statistics 19. Nonparametric statistical methods were used. Continuous variables were expressed as median and interquartile range (IQR) if not indicated otherwise. Univariate comparisons of two independent groups were done using the Mann–Whitney-U test or Chi-Square/Fisher’s exact test. Confirmatory analyses relative to >2 groups were performed with the Kruskal–Wallis test followed by post hoc testing via Mann–Whitney U test with Bonferroni adjustment for multiple testing.

## Results

### Normal prevalence of polymorphisms for COMT, CRHR1 and FKBP5 in CFS

First, we analyzed 74 CFS patients and 76 healthy controls for their genotypic and allelic frequencies for the SNPs rs4680 for COMT, rs1360780 for FKBP5, and rs12944712 for CRHR1, respectively. Characteristics of patients including the Bell score as a level of mental and physical disability score from 100 (healthy) to 0 (severe disease) are described in Table 1 and distribution of SNPs in Table 2. We found no difference between CFS patients and controls in the prevalence of the COMT SNP rs4680, as well as a similar distribution of FKBP5 SNP rs1360780 and CRHR1 SNP rs12944712, respectively.

**Table 1:** Characteristics of CFS and control group

	Healthy (n = 76)	CFS (n = 74)
Age (years)	40 ± 17	40 ± 9
m/f (%)	43/57	37/63
Bell score	na	30 ± 10

*na* not applicable.

**Table 2:** Association of SNPs with CFS

SNP	n	Minor −/−	Hetero −/+	Major +/+	Genotypic frequencies (%)			Allelic frequencies (%)		p value
CRHR1 rs12944712					*Met/Met*	*Met/Val*	*Val/Val*	*G*	*A*	
Healthy	76	26	38	12	0.34	0.50	0.16	0.59	0.41	0.6189
CFS	74	27	34	13	0.36	0.46	0.18	0.59	0.41	
COMT rs4680					*G/G*	*G/A*	*A/A*	*G*	*A*	
Healthy	76	18	45	13	0.24	0.59	0.17	0.53	0.47	0.7439
CFS	74	22	37	15	0.30	0.50	0.20	0.55	0.45	
FKBP5 rs1360780					*T/T*	*T/C*	*C/C*	*T*	*C*	
Healthy	76	8	29	39	0.11	0.38	0.51	0.30	0.70	0.6396
CFS	74	5	33	36	0.07	0.45	0.49	0.29	0.71	

Allelic discrimination PCR was performed for 74 CFS patients and 76 healthy controls.

### Enhanced cortisol levels associated with the hypofunctional Met variant of *COMT* in CFS

**Fig. 1:**
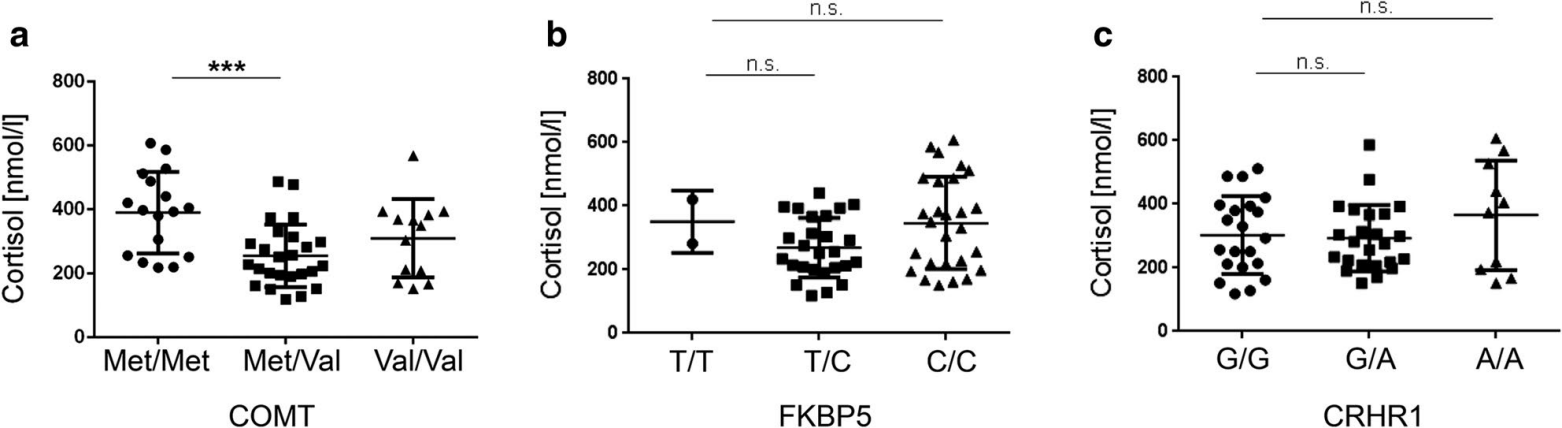
Cortisol levels in CFS patients. Serum of 55 CFS patients was analyzed for cortisol levels. Patients were grouped in the respective haplotypes of the SNPs for **a** rs4680 for COMT, **b** rs1360780 for FKBP5, and **c** rs12944712 for CRHR1. Statistic analysis was performed with the Kruskal–Wallis test followed by post hoc testing via two-tailed Mann–Whitney U test with Bonferroni adjustment for multiple testing with ***p < 0.00033 (0.001/3 comparisons) as significant, *ns* not significant.

To assess the functional relevance of the SNPs, cortisol levels were compared in the groups of CFS patients with wild type, heterozygous and homozygous variants. We found significantly enhanced cortisol levels in the group of CFS patients with the Met/Met allele compared to heterozygous and Val/Val allele carrying patients (Fig. 1a). In contrast no association was found for cortisol levels and SNPs for FKBP5 and CRHR1 (Fig. 1b, c).

### SNPs for COMT and FKBP5 are associated with immunoglobulin levels in CFS

**Fig. 2:**
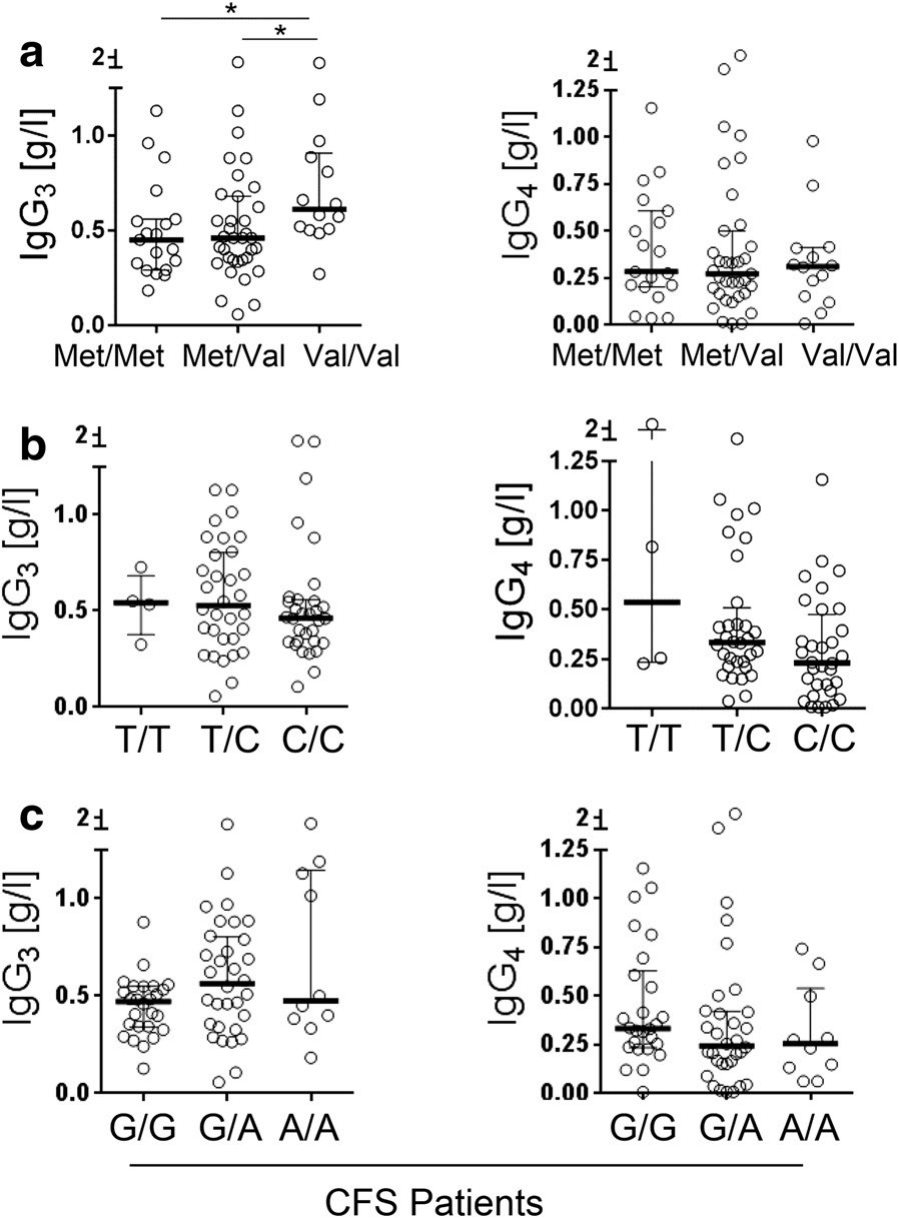
IgG_3_ and IgG_4_ levels in CFS patients with COMT, FKBP5, and CRHR1 SNP. **a** Levels of immunoglobulin subclasses IgG_3_ and IgG_4_ were determined in serum of CFS patients and grouped according to their genotype for rs4680 for COMT, **b** rs1360780 for FKBP5, and **c** rs12944712 for CRHR1, respectively. Statistic analysis was performed with the Kruskal–Wallis test followed by post hoc testing via two-tailed Mann–Whitney U test with Bonferroni adjustment for multiple testing with *p < 0.017 (0,05/3 comparisons) as significant.

We had observed that a subset of CFS patients has diminished IgG_3_ and IgG_4_ levels. Due to the known effect of stress and cortisol on immunoglobulin levels [30, 31] we therefore correlated the haplotypes for the SNPs for COMT, FKBP5 and CRHR1 with the patient’s IgG_3_ and IgG_4_ levels. Interestingly, we found that the homozygous and heterozygous Met variant of COMT rs4680 is associated with significantly lower IgG_3_ levels in comparison to the homozygous Val genotype (Fig. 2a). No difference for the other IgG subclasses as well as IgG, IgM, and IgA was observed (data not shown). Oppositely, in patients with the FKBP5 SNP rs1360780 wild type variant C/C significantly lower levels of IgG_4_ were found compared to the C/T or T/T variant, but no differences in the IgG_3_ levels (Fig. 2b). However, the differences in IgG_4_ were no longer significant after Bonferroni correction. No differences were found for the CRHR1 SNP rs12944712 (A/A, A/G, G/G) and IgG_3/4_ levels (Fig. 2c).

**Fig. 3:**
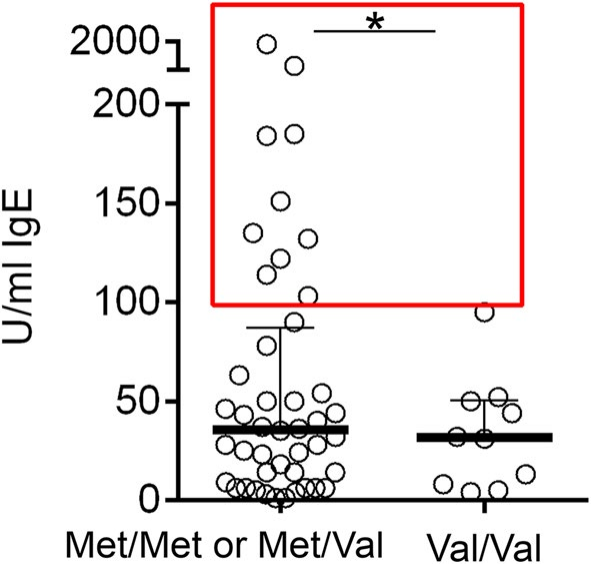
Correlation of IgE levels with COMT SNP rs4680. Serum of 54 CFS patients was analyzed for IgE. Patients were group in either Met/Met, Met/Val, or the wild type variant Val/Val. Enhanced IgE levels are defined >100 U/ml (*dashed line*). Statistic analysis was performed with two-tailed Chi-Square/Fisher’s exact test with *p < 0.05 between Met/Met or Met/Val and the variant Val/Val.

Furthermore, it had been shown that norepinephrine upregulates IgE production [32]. Thus, we compared COMT subgroups in 54 CFS patients whose IgE levels had been determined. Indeed, we found that all 10 patients with enhanced IgE levels, defined as above 100 U/ml, had hetero- or homozygous Met allele (n = 10/44, 22.7 %) whereas no patient (n = 0/10) was found in the Val/Val group (Fig. 3). Of those 10 patients with elevated IgE, 7 patients had reported allergies.

### COMT SNP rs4680 is associated with recurrent infections in CFS

**Fig. 4:**
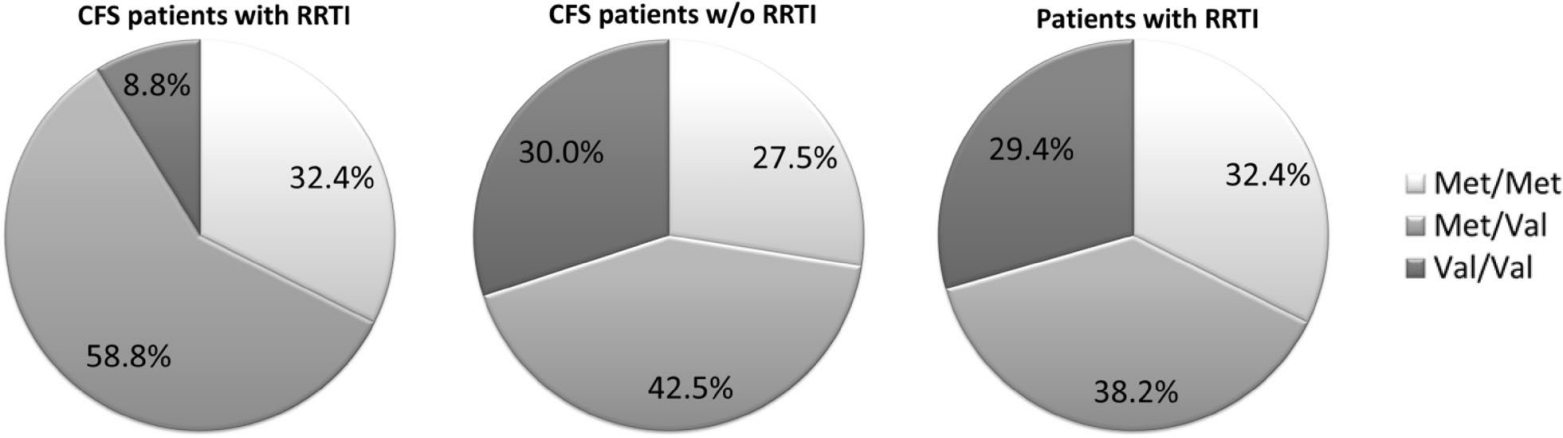
Distribution of variants for COMT rs4680 in 34 CFS patients with RRTI and 40 CFS patients without RRTI. As control 68 non-CFS patients with RRTI were analysed. Statistic analysis was performed with two-tailed Chi-Square/Fisher’s exact test with *p < 0.05 between the variant Met/Met and Met/Val, and the major variant Val/Val.

As a subset of patients with CFS suffers from susceptibility to infections, we analyzed if COMT SNPs are associated with infections. 34 of the 74 patients reported to suffer from RRTI. The clinical characteristics of the patient cohort are shown in Table 3. 47 % of CFS patients with RRTI had received antibiotic treatment for RRTI. There was no significant difference in median ages, gender or Bell score between the two groups with and without RRTI. Interestingly, we found that RRTI are significantly more frequent in the presence of the Met variant of SNP rs4680 for COMT in the CFS patients (Fig. 4, p = 0.023). While 31 (91 %) of the 34 patients with RRTI had Met, 28 of 40 (70 %) patients without RRTI had a Met allele. In contrast, no association was observed for the SNPs for FKBP5 rs1360780 and CRHR1 rs12944712.

**Table 3:** Characteristics of CFS and non-CFS patients with RRTI

	CFS w/o RRTI (n = 40)	CFS with RRTI (n = 34)	non-CFS RRTI (n = 68)
Age (years)	39 ± 10	42 ± 10	38 ± 13
m/f	37/63 %	35/65 %	29/71 %
Bell score	40 ± 10	30 ± 10	n.a.
RRTI (n)		34 (46 %)	68 (100 %)
Lower RRTI (n)		5 (15 %)	30 (44 %)
Pneumonia		9 %	19 %
Bronchitis		15 %	31 %
Upper RRTI (n)		34 (100 %)	54 (79 %)
Sinusitis		79 %	57 %
Pharyngitis		15 %	16 %
Tonsilitis		6 %	10 %

*na* not applicable.

**Fig. 5:**
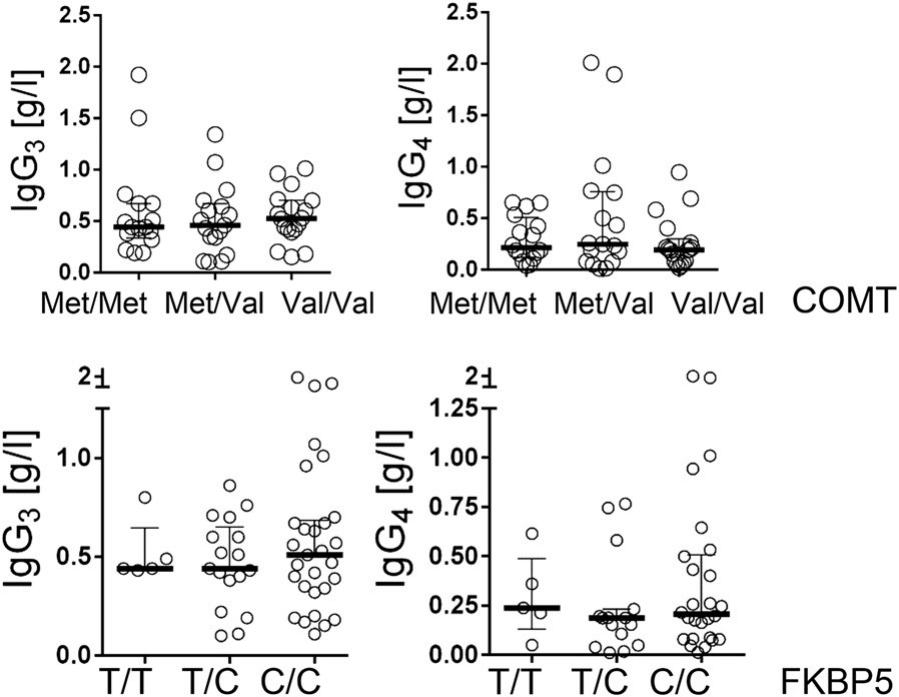
IgG_3_ and IgG_4_ levels in 68 non-CFS patients with RRTI with COMT and FKBP5 SNP. Statistic analysis was performed with the Kruskal–Wallis test followed by post hoc testing via two-tailed Mann–Whitney U test with Bonferroni adjustment for multiple testing.

To study if this association of Met with RRTI was seen in non-CFS patients as well we analyzed an additional cohort of 68 patients, who had presented due to RRTI at our outpatient clinic (patient characteristics shown in Table 3) for the prevalence of these COMT variants. In this cohort the frequency of the Met variant was similar to CFS patients without RRTI (71 %). Further, neither an association of COMT variants with IgG_3_ and IgG_4_ levels (Fig. 5) nor with IgE levels (not shown) was found.

## Discussion

In this study, we analyzed polymorphisms of genes regulating the metabolism of neurotransmitters and cortisol in CFS patients. We found no difference in the prevalence of the SNPs for COMT rs4680, FKBP5 rs1360780 and CRHR1 rs12944712 in CFS patients compared to controls. However, we observed elevated IgE levels, diminished IgG_3_ levels and an enhanced susceptibility to RRTI in patients with the hypofunctional Met variant for COMT SNP rs4680. Further, diminished IgG_4_ levels were associated with the wild type allele of the SNP rs1360780 in comparison to the variant of FKBP5.

First of all, our observation of similar frequency of the COMT SNP in CFS patients and controls is in contrast to previous reports. A study of Sommerfeldt et al. showed a higher prevalence of the COMT SNP rs4680 in adolescent CFS patients [22]. In contrast, our patients fulfilling Fukuda and Canadian criteria were at least 18 years old. For our healthy control group, we reached allelic frequencies with 0.59 for the G variant and 0.41 for the A variant of the COMT SNP that are comparable to data supplied by the NCBI dbSNP Short Genetic Variations Database that quote 0.64 for the G and 0.36 for the A variant. Another study postulated SNPs for COMT and CRHR1, identified by a calculation model, as predictive biomarkers for CFS [23]. Restrictively, this analysis included five different SNPs for COMT and three SNPs for CRHR1, with the SNPs rs4680 and rs13060780 that were analyzed in our study not present in the study of Goertzel et al.

A focus in our work was set on the analysis of the hypofunctional Met variant of COMT that is linked to a diminished degradation of catecholamines leading to enhanced norepinephrine and cortisol. Enhanced cortisol levels were previously shown in healthy subjects with the hypofunctional Met/Met variant of COMT rs4680 [33–35]. In line with this, we observed significantly higher cortisol levels in CFS patients with the COMT haplotype Met/Met compared to the Val haplotypes. Also polymorphisms in FKBP5 and CRHR1, both regulating the cortisol response to stress, were associated with changes in HPA axis reactivity [27, 36]. However, in these studies the genotypes were not correlated to cortisol or norepinephrine levels. No significant difference in cortisol was found for FKBP5 and CRHR1 in our patients.

Inflammation is regulated by neuroendocrine hormones including glucocorticoids and catecholamines [37]. Immune cells are known to express receptors for both cortisol and norepinephrine [38, 39]. In COMT deficient mice minor changes mainly in male animals were found with total number of T-, and B-cells and T-cell proliferative response decreased, but no impact on NK cell cytotoxicity, oxygen radical production and immunoglobulin production [40]. However, the influence of stress and infections on the immune function was not studied in this model. In a comprehensive study of immune parameters in CFS patients we had observed diminished immunoglobulin subclass levels in 25 % of CFS patients predominantly of IgG_3_ and IgG_4_ (Guenther et al. unpublished). Further we found an association of IgG_3_ deficiency and susceptibility to RRTI. Therefore, we were interested to see if there is an association of the COMT SNP rs4680 Met variant with immunoglobulin levels and RRTI. Lower levels of IgG_3_ but not of the other immunoglobulins were found in CFS patients with the Met variant. The half-life of IgG is dependent on the neonatal FcRn, which recycles and protects IgG from degradation. IgG_3_ has a shorter half-life as the other IgGs which is due to the lower affinity of IgG_3_ to FcRn [41]. Steroids decrease FcRn expression and function [42]. Thus, due to its shorter half-life IgG_3_ may be more susceptible to the suppressive effects of steroids. We could also observe that the Met variant is associated with enhanced susceptibility to RRTI. Interestingly, we found no enhanced frequency of Met alleles in a group of patients with RRTI without CFS. One possible explanation for this finding is that the dysregulation of cortisol production and immune cell sensitivity to steroids described in CFS patients [10, 17–19] makes CFS patients with the Met variant more susceptible to the immunosuppressive effect of cortisol.

In line with our data of lower IgG_3_ levels in CFS patients with the Met/Met genotype of COMT in patients with fibromyalgia this COMT variant was shown to be associated with lower salivary IgA concentration [24]. Further, in fibromyalgia enhanced pain sensitivity was reported for patients with this COMT variant [43]. No association with muscle and joint pain or headache or the Bell score was found with the Met variant in our CFS patients.

Further we found that CFS patients with the Met variant had higher IgE levels. As norepinephrine was shown to upregulate the production of IgE [32] the assumed stress-related diminished degradation of norepinephrine in patients with the Met/Met haplotype may result in enhanced IgE levels.

FKBP5 expression is induced by steroids [44, 45] and the T variant of rs1360780 was shown to result in enhanced negative feed-back of the cortisol response as shown in the dexamethasone/CRH test [28]. This SNP was found to be associated with increased risk for posttraumatic stress [46] and depression [47]. We could not find a correlation of the FKBP5 SNP with cortisol levels. We observed, however, that the wild type C allele of the FKBP5 SNP that has lower repressive function and may thus result in higher cortisol levels is associated with a trend of lower IgG_4_ levels in our patients.

Limitations of the study are the relatively small sample size for a SNP analysis and that clinical parameters were evaluated retrospectively.

## Conclusion

In conclusion, our data provides evidence for an association of the hypofunctional COMT SNP with immune function in CFS. Both enhanced catecholamine and cortisol levels may lead to diminished IgG production.

## Abbreviations

CFS: chronic fatigue syndrome
CNS: central nervous system
COMT: catechol-O-methyltransferase
CRHR1: corticotropin-releasing hormone receptor 1
FKBP5: FK506 binding protein 5
HPA axis: hypothalamic–pituitary–adrenal axis
Ig: immunoglobulin
RRTI: recurrent respiratory tract infections
SNP: single nucleotide polymorphism
